# Drug Shortages From the Perspectives of Authorities and Pharmacy Practice in the Netherlands: An Observational Study

**DOI:** 10.3389/fphar.2018.01243

**Published:** 2018-10-31

**Authors:** Doerine J. Postma, Peter A. G. M. De Smet, Christine C. Gispen-de Wied, Hubert G. M. Leufkens, Aukje K. Mantel-Teeuwisse

**Affiliations:** ^1^Royal Dutch Pharmacists Association, The Hague, Netherlands; ^2^Division of Pharmacoepidemiology and Clinical Pharmacology, Utrecht Institute for Pharmaceutical Sciences, Utrecht University, Utrecht, Netherlands; ^3^Departments of Clinical Pharmacy and IQ Healthcare, Radboud Institute for Health Sciences, Radboud University Medical Center, Nijmegen, Netherlands; ^4^Medicines Evaluation Board, Utrecht, Netherlands

**Keywords:** drug shortages, authorities, pharmacy practice, signals, observational study

## Abstract

**Background:** Drug shortages are a potential threat to public health. Reliable data on drug shortages is limited. The objective was to examine the extent and nature of potential drug shortages signaled by authorities and pharmacy practice in the Netherlands

**Materials and Methods:** The primary working systems of Dutch authorities (Medicines Evaluation Board and Health and Youth Care Inspectorate) and the archives of pharmacy practice (Royal Dutch Pharmacists Association) from 2012 to 2015 were searched for number, characteristics, overlap, and date of signals on potential drug shortages. Also, the product characteristics of the potential drug shortages were analyzed from the two different sources

**Results:** Authorities detected 2.6 times more signals on potential shortages than pharmacy practice. Only 438 (8%) out of 5,731 potential drug shortages were detected by both authorities and pharmacy practice. Signals were detected later by authorities than by pharmacy practice, especially on potential permanent shortages (median difference −180 days (IQR: −4 to −405 days)). Authorities detected by majority (72%) signals related to permanent shortages with relative overrepresentation of rectal products and anti-infectives for systemic use. In contrast, pharmacy practice detected by majority (71%) signals related to temporary shortages with relative overrepresentation of ocular and cutaneous products, anti-infectives for systemic use, products for sensory organs and dermatologicals.

**Conclusions:** Authorities and pharmacy practice detected different signals on potential drug shortages with little overlap. Combining data from both authorities and pharmacy practice seems to be necessary in order to gain a more complete overview and maximum insight in potential drug shortages at a national level. Moreover, the finding that authorities were informed later than pharmacy practice causes concerns in terms of opportunities for authorities to assist pharmacy practice to find solutions for shortages.

## Introduction

Drug shortages are a potential threat to public health. According to the World Health Organization, drug shortages are becoming, if not are already, a global problem (Gray and Manasse, 2012; WHO, 2016). Countries all over the world have reported drug shortages and subsequent problems that affect both developing and developed countries (Lynas, 2013; Quilty, 2014; Chen et al., 2016; Rosa et al., 2016; De Weerdt et al., 2017). Shortages have resulted in problems with medication such as errors, rationing, delayed supply, and denial of treatment in some cases (Fox et al., 2014; Vail et al., 2017). Shortages not only impact patients; health care professionals are confronted with lack of information on shortages and need to find proper solutions, causing pressure on primary and hospital care (McCartney, 2015), as well as an increase in costs (Iacobucci, 2017). Reliable data on drug shortages is limited. Scientific publications are mainly based on narrative reviews (De Weerdt et al., 2017), survey results (Pauwels et al., 2015), or anecdotal evidence from studies of specific drugs (Malone et al., 2016). The growing problems due to shortages have been addressed in public media as well (Koba, 2015; Fink, 2016). The media has especially focused on the harmful effects of shortages for patients.

When shortages occur, authorities struggle with whether and how to intervene. In the United States, the Food and Drug Administration (FDA) has received new and expanded regulatory powers to respond to national shortages by the Food and Drug Administration Safety and Innovation Act in 2011 (McCarthy, 2013). According to this act, a marketing authorization holder (MAH) has to notify the FDA of a discontinuation or interruption when it will lead to a meaningful supply interruption of a drug that is life-supporting, life-sustaining, or intended for use in the prevention or treatment of a debilitating disease or condition. They have to notify the FDA at least six months in advance or as soon as possible, but not later than five business days after the start of discontinuation or interruption. If an MAH fails to submit this information, letters are made public on the website of the FDA (Food and Drug Food Drug Administration, 2015). It seems that these measures have had impact. The number of shortages in the United States decreased by 24% in 2012 and by 60% in 2013, respectively (Fox et al., 2014). European legislation states that “*the holder of a marketing authorization for a medicinal product … shall, within the limits of his responsibilities, ensure appropriate and continued supplies of that medicinal product to pharmacies*” (The European Parliament the Council of the European Union, 2011). The European Medicines Agency has been involved in several drug shortages, in case the shortage was linked to a safety concern and multiple Member States were affected. However, most drug shortages are handled at a national level by national competent authorities (European Medicines Agency, 2013). In France, the French legislator chose to introduce new measures to the law in January 2016 to fight drug shortages. MAHs are now legally bound to ensure sustained supply and stocks of drugs they market in France to meet needs of patients (Bocquet et al., 2017). In the Netherlands, the Medicines Act of 2007 states that “*a marketing authorization holder takes care that a medicinal product with a marketing authorization is in stock in sufficient amount for wholesalers and pharmacists*” and the MAH “*must notify the authorities of interruption of a medicine. Foreseen shortages must be reported 2 months in advance and unforeseen as soon as possible*” (Dutch Government, 2007). Despite this legislation, pharmacies face drug shortages regularly, which makes it important to monitor these shortages at a national level and to advise on potential solutions. The Royal Dutch Pharmacists Association (KNMP) launched a public website to inform pharmacists about new shortages and about the status of ongoing shortages in 2004. Similar initiatives have been launched inside (Bochenek et al., 2017) and outside the EU. The University of Utah's Drug Information Service (UUDIS), for example, has a comparable website which is hosted by the American Society of Health-System Pharmacists (ASHP) (Fox et al., 2009).

All these initiatives to monitor and manage drug shortages, legally bounded or not, show the critical importance of timely and high-quality information on potential shortages throughout the supply chain. Only when signals on potential drug shortages are tagged in an efficient and adequate manner, authorities and health care professionals can act accordingly. This study aims to evaluate the added value of signals on potential shortages derived from two different sources: authorities and pharmacy practice. Therefore, we examined the extent and nature of potential drug shortages signaled by authorities and pharmacy practice in the Netherlands.

## Methods

### Data sources

We collected data on potential drug shortages in 2012–2015 from the Dutch authorities and pharmacy practice. Authorities have received mandatory reports from MAHs since the Medicines Act of 2007. MAHs have to report foreseen shortages to the Dutch Medicines Evaluation Board (MEB) 2 months in advance and unforeseen shortages to the Dutch Health and Youth Care Inspectorate (Inspectorate) as soon as possible. We retrieved data from both of these authorities, thereby excluding duplicates.

Pharmacists, both hospital-based and community-based pharmacists, have voluntarily reported shortages to the Royal Dutch Pharmacists Association (Koninklijke Nederlandse Maatschappij ter bevordering der Pharmacie, KNMP) since 2004. These reports from pharmacists best reflect shortages experienced in pharmacy practice. Other professionals and patients can also report, although this occurs less frequently. MAHs can also report shortages here but are not obliged to do so. We retrieved data from pharmacy practice from the archives of the KNMP. KNMP publishes the information, after validating with the MAH, on a public website (farmanco.knmp.nl) when the medicinal product is nationally unavailable for at least 2 weeks. KNMP also advises on potential solutions, which were publicly available during the study period.

### Definitions

We defined a signal on a potential shortage at the level of products with a marketing authorization (MA) for human use thus excluding veterinary drugs. Homeopathic and herbal drugs were also excluded as well as unregistered products such as raw materials, pharmacy preparations and food additives. We excluded signals on parallel import products since parallel importers are not legally obliged to notify the authorities on potential shortages. We defined a signal to be overlapping by two criteria. First, the signal was related to the same MA. Second, for a potential temporary shortage the signal had to be detected by authorities 30 days before or after the signal was detected by pharmacy practice. For a potentially permanent shortage the signal had to be detected by both authorities and pharmacy practice at any point during the study period.

### Data analysis

We analyzed the signals on potential shortages per calendar year, distinguishing between signals on permanent shortages (withdrawals) and temporary shortages (supply interruptions). When products were permanent in shortage after being temporary in shortage, we considered this a permanent shortage. We also distinguished between signals on marketed and not marketed products in the Netherlands based on the national drug database (G-Standaard) (Z-index^1^). Every medicinal product that is actually marketed is listed in this database. We analyzed the overlap in signals between authorities and pharmacy practice. For overlapping signals on potentially permanent shortages, we plotted the date the shortage signal was detected by authorities vs. the date it was detected by pharmacy practice. Signals on temporary shortages were excluded from this latter analysis, since we defined a time frame as part of the definition for overlap. We calculated the median difference in time, as well as the interquartile range (IQR). All marketed products which were signaled for a potential shortage, were categorized by their route of administration according to the Standard Terms of the EDQM (European Directorate for the Quality of Medicines HealthCare, 2016) and by their Anatomical Therapeutic Chemical (ATC) class of the WHO (WHO Collaborating Centre for Drug Statistics Methodology ATC - Structure, 2018). These categories were compared to the overall characteristics of the products on the Dutch market (data obtained from G-Standaard).

## Results

**Figure 1:**
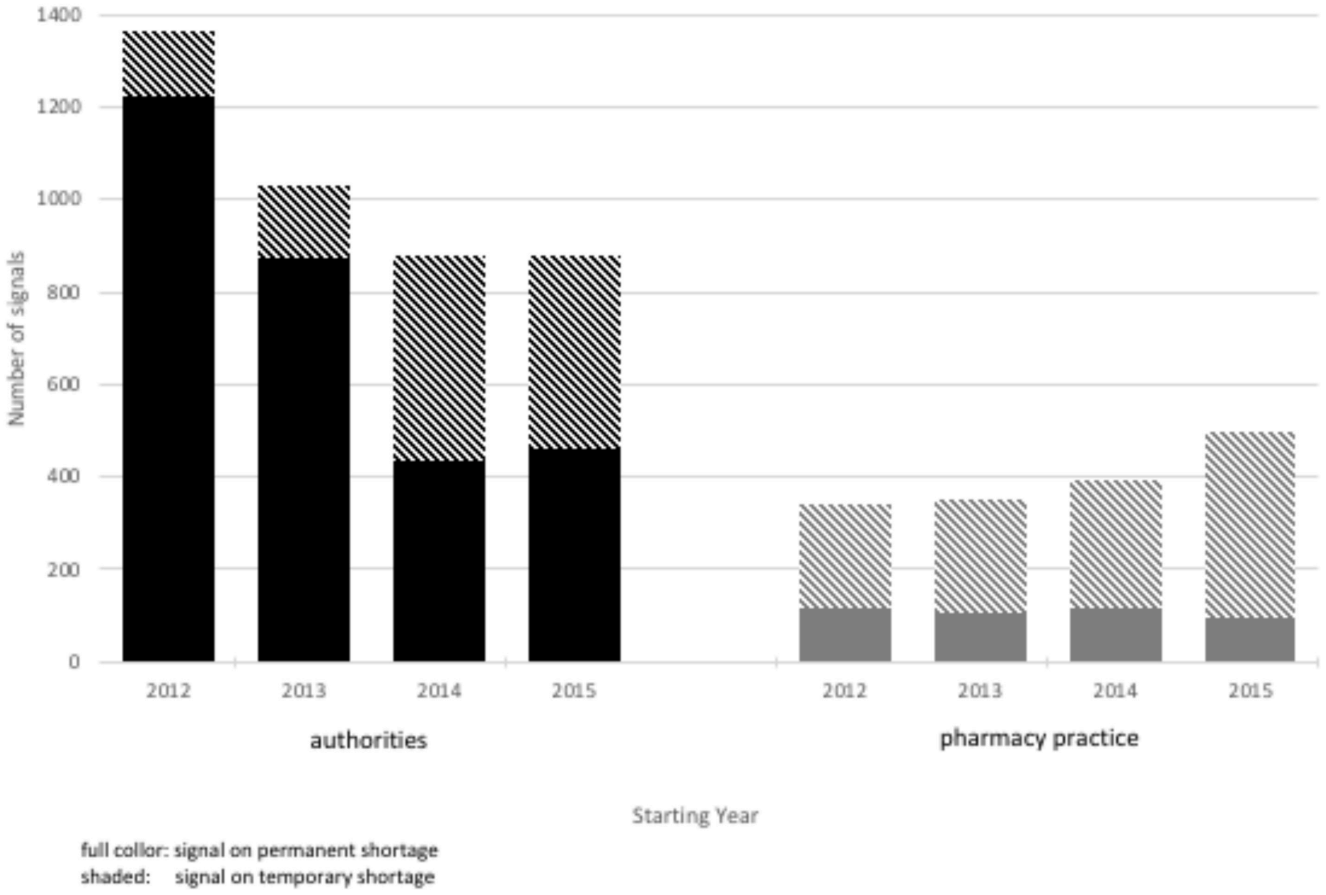
Number of new signals on potential shortages per calendar year.

**Figure 2:**
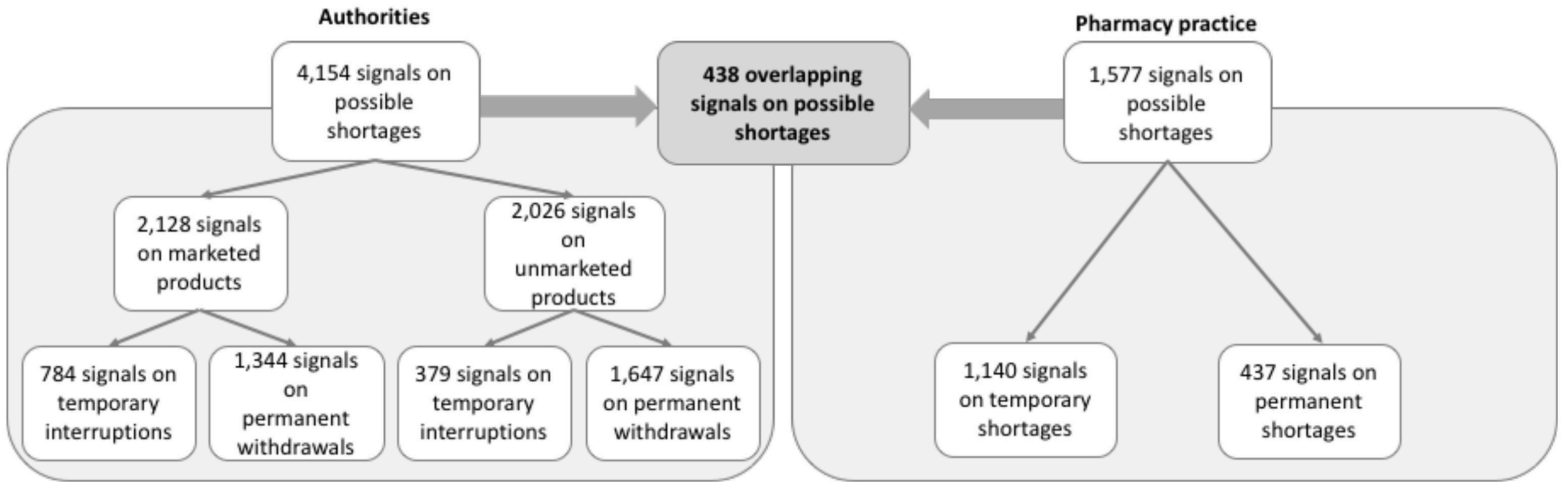
Overlap in signals on potential shortages between authorities and pharmacy practice.

Authorities detected signals on 4,154 potential shortages; 2,128 (51%) signals were related to products marketed in the Netherlands. Most signals (*n* = 2,991; 72%) were on permanent shortages. The overall number of signals to authorities decreased during the study period from 1,366 to 880 per year (Figure 1), despite an increase in the number of signals on temporary shortages. Pharmacy practice detected signals on 1,577 potential shortages (Figure 2). Most signals (*n* = 1,140; 72%) were for temporary shortages. The overall number of signals to pharmacy practice increased during the study period from 340 to 495 per year due to an increase in signals on temporary shortages (Figure 1). During the study period, authorities detected in total 2.6 times more signals on potential shortages than pharmacy practice.

**Figure 3:**
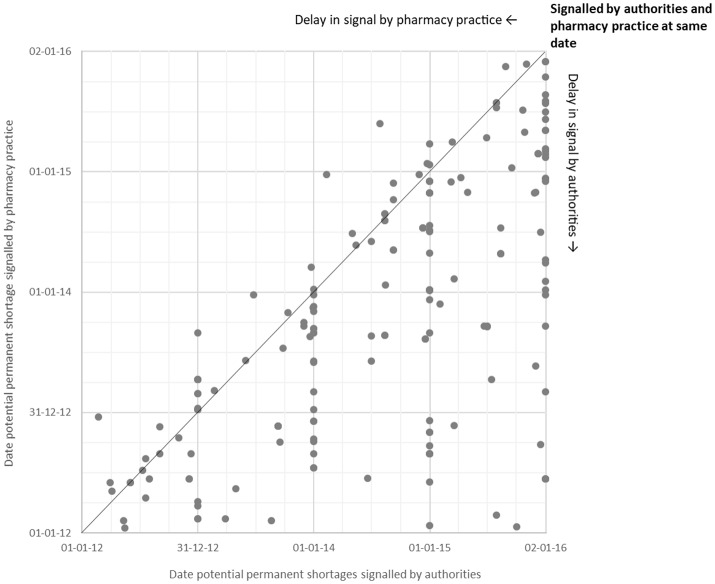
Date of signal by authorities vs. pharmacy practice for potential permanent shortages.

Of a total of 5,731 signals on potential shortages detected by both authorities and pharmacy practice during the study period, only 438 (8%) overlapped (Figure 2). Overlapping signals were for temporary shortages (*n* = 251; 57%) and for permanent shortages (*n* = 187, 43%). The median difference in reporting dates for permanent shortages between authorities and pharmacy practice was −180 days (interquartile range [IQR]: −8 to −405 days), meaning that pharmacy practice detected the overlapping signals earlier than authorities (Figure 3). Overlapping signals on temporary shortages were also detected by pharmacy practice before they were detected by authorities, with a median difference of −1 day (interquartile range [IQR]: 23 to −203 days).

### Characteristics of signals

**Figure 4:**
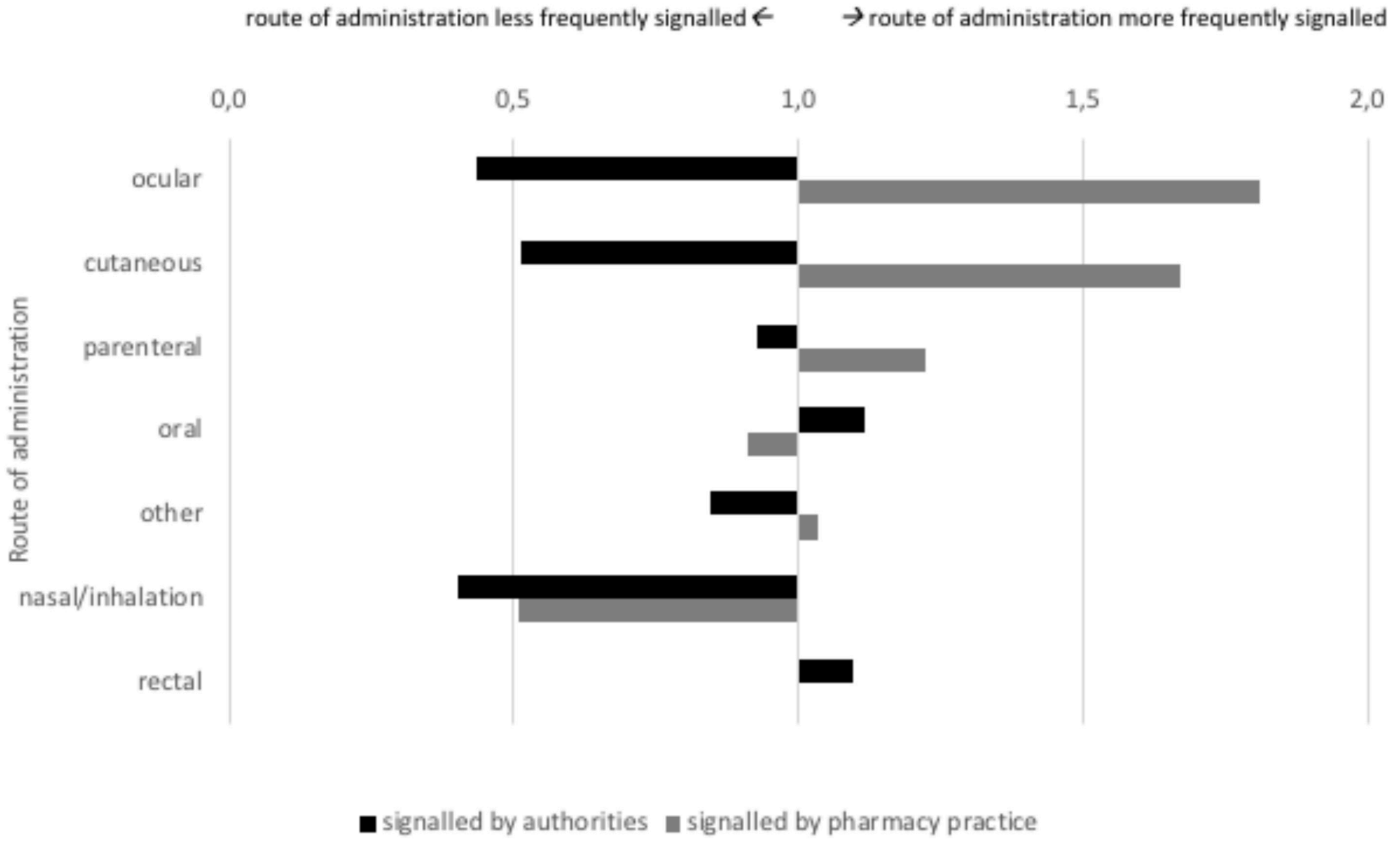
Signals on potential shortages characterized by route of administration (indexed for market share; at 1.0 the proportion of signaled products with this route of administration is equal to the proportion of products on the Dutch market with this route of administration.

**Figure 5:**
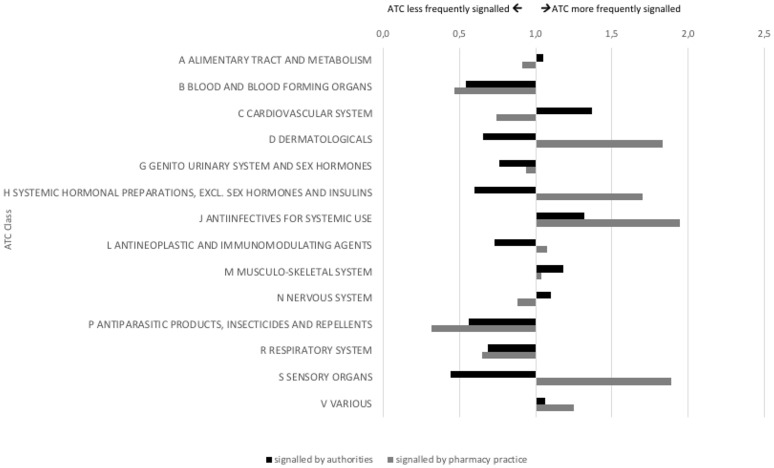
Signals on potential shortages characterized by ATC class (indexed for market share; at 1.0 the proportion of signaled products with this ATC class is equal to the proportion of products on the Dutch market with this ATC class).

The products for which signals were detected by authorities and pharmacy practice differed in route of administration and showed partial similarity regarding their ATC class (Figures 4, 5). Authorities detected signals more frequently for rectal products (1.2 times as compared to overall market share) and anti-infectives for systemic use (1.4 times). Pharmacy practice detected signals more frequently for ocular (1.8 times) and cutaneous (1.7 times) products and anti-infectives for systemic use (2.1 times), products for sensory organs (1.8 times), and dermatologicals (1.6 times).

## Discussion

Our results show totally different perspectives on potential drug shortages when signals from authorities and pharmacy practice are compared. Comparing both sources, we found a very small overlap in signals (8%), different types and characteristics of the affected products and a differential timing of signals. Whereas authorities faced a decrease in number of signals over time, pharmacy practice faced an increase in number of signals. For authorities, a typical signal as identified in this study would be the permanent shortage of a rectal product or an anti-infective for systemic use. For pharmacy practice, a typical signal would be a temporary shortage of an ocular product or an anti-infective for systemic use. Signals to authorities were likely detected by pharmacy practice as well, but the latter detected it earlier. Signals to pharmacy practice may have been detected by authorities as well, but this would not be very likely.

Authorities detected signals on permanent shortages much later than pharmacy practice. Although signals on temporary shortages were detected with a median difference of 1 day, results show a larger variation toward the delay to authorities (Q_3_ = −203 days) than delay to pharmacy practice (Q_1_ = −23 days). Since early signaling to authorities is considered critical in preventing and mitigating a drug shortage (Fox et al., 2014), authorities were insufficiently able to intervene during problems in pharmacy practice. Combining both databases gives much more insight and potential to timely action.

The major strength of this study is the direct comparison between signals on potential shortages to authorities and to pharmacy practice for a study period of several years. Authorities in many countries may have data on drug shortages but only few countries collect data from pharmacy practice at a national level and have historical data. It could be argued that data from pharmacy practice mainly relate to local shortages. However, 90% of these signals in our study were confirmed by the MAH as a shortage at a national level. The best available data on drug shortages originate from the American UUDIS/ASHP on pharmacy practice and from the FDA authority (Fox et al., 2009, 2014). These data are comparable to the datasets in the Netherlands since the Dutch as well as the American data report shortages at a national level which have been confirmed by MAHs (American Society of Health-System Pharmacists, 2018; Food Drug Administration, 2018). A direct comparison between the American databases on shortages was only made for a short study period, January 2013 through March 2013. FDA and UUDIS identified a different number of shortages; 17 and 39 respectively. Only 8 shortages overlapped and 5 of these 8 shortages were signaled 6 to 50 days earlier by FDA than UUDIS (US Government Accountablility Office, 2016). UUDIS and FDA have different perspectives on the definition of a shortage, organizations' objectives and the data at their disposal, resulting in different numbers (Wosinska and Jensen, 2015; US Government Accountablility Office, 2016). The UUDIS found (Chen et al., 2016) that 55% of their shortages affected parenteral products and the most common classes of drugs were central nervous system agents (17%) and anti-infective agents (14%). These latter results differ from our findings, which may be explained by the fact that we studied signals on potential shortages, whereas UUDIS studied shortages. Also, shortages reported to the UUDIS are published on a website which is hosted by the ASHP which represents pharmacists who serve in hospital settings. As expected UUDIS signals relatively more hospital products. KNMP detected signals from hospital-based and community-based pharmacists.

This study clearly shows a difference in signals on potential shortages detected by authorities and pharmacy practice. Half of this gap (2,026 signals; 51%) is due to signals on products which were never marketed in the Netherlands and therefore could not affect pharmacy practice. Another part of this gap is probably explained by the different sources of signals, perspective of authorities and pharmacy practice and their definition of a shortage. Authorities detect signals from MAHs whereas pharmacy practice detects signals from pharmacists. MAHs may be reluctant to signal potential drug shortages due to logistical and marketing reasons, since potential shortages might disrupt market shares and could be regarded as a negative message to communicate. Not reporting of mandatory signals has not been sanctioned by authorities so far, since they have been focusing primarily on finding solutions for shortages and not on reporting (Dutch Government, Ministry of Health, Welfare and Sport, 2017). Pharmacists may be reluctant to signal potential drug shortages in view of the voluntary nature of the system. Pharmacists probably signaled potential shortages with a large practical impact (to be solved by replacing with a different active substance) more frequently. Potential shortages with less impact (to be solved by replacing with a product with the same therapeutic active substance) were probably signaled less frequently.

Whereas authorities focus on shortages with patient impact at the population level, pharmacy practice handles continuity of care at the patient level. It is known that the definition of a drug shortage depends on the type of organization (De Weerdt et al., 2015) and can be interpreted differently (Barlas, 2014). In addition, not all signals become a shortage, and the impact of a shortage may differ between products and between stakeholders. Further research is needed to provide more insight into which signals have led to a shortage as well as the impact of these shortages at population and individual levels.

Two recent developments may narrow the observed information gap. First, the definition of a drug shortage recently became more uniform for MAHs. At an European level, MAHs together with all other organizations in the pharmaceutical supply chain agreed in 2016 on the definition of a suspected drug shortage as: “*the inability of a community or hospital pharmacy, as a result of factors beyond their control, to supply a medicinal product to a patient within a defined period, for example 72 h*” (AESGP et al., 2017. Also, Dutch authorities agreed on a broader definition of a shortage in 2017: “*an interruption of availability of a medicinal product that is a burden for patients and where the burden is greater than is the case for regular (generic) substitution*” (Dutch Government, Ministry of Health, Welfare and Sport, 2017). Both agreements may lead to more similarity in signals to authorities and to pharmacy practice. Second, MAHs can report to the newly established Medicine Shortages and Defects Notification Center of the Dutch authorities since 2017. This center enables MAHs to report at a central point, therefore providing a quicker and more complete overview to authorities. Future research is needed to examine the impact of these developments.

## Conclusions

Authorities and pharmacy practice were informed on drug shortages differently with little overlap. In order to gain maximum insight in drug shortages at a national level combining data from both authorities and pharmacy practice seems to be necessary. No single source can give a complete picture. Moreover, the finding that authorities were informed later than pharmacy practice, raises concerns in terms of opportunities for authorities to assist practice to sort out solutions for drug shortages.

## Data availability

The datasets for this manuscript are not publicly available, since data is related to specific products and manufacturers, which is confidential information. No additional data available.

## Author contributions

All authors were involved in the design of the original protocol and amending the protocol. Data entry from authorities was carried out by the Inspectorate and MEB and from pharmacy practice by KNMP. DP extracted and cleaned the data and analyzed the data with input from CG and PDS. DP wrote the first draft of the manuscript with AM. All authors contributed to subsequent and final drafts. AM is guarantor of the paper.

### Conflict of interest statement

The authors declare that the research was conducted in the absence of any commercial or financial relationships that could be construed as a potential conflict of interest.

## Footnotes

^1^Z-index About Z Index (2018). [Online]. Available: https://www.z-index.nl/english [Accessed July, 2018]

## References

[B1] AESGP EAHP, EAEPC, EFPIA, EIPG, GIRP, Medicines for Europe PGEU (2017). Joint supply chain actors statement on information and medicinal products shortages. Available online at: http://www.pgeu.eu/en/press/239:pr-joint-statement-on-shortages.html (Accessed July 2018).

[B2] American Society of Health-System Pharmacists (2018). FDA and ASHP Shortage Parameters. Available online at: https://www.ashp.org/Drug-Shortages/Current-Shortages/FDA-and-ASHP-Shortage-Parameters (Accessed July, 2018).

[B3] Barlas S. (2014). Manufacturers and hospitals spar over drug shortage reporting: FDA proposal seeks to improve early warning system. Pharm. Ther. 39, 152–215. 24790393 PMC4005123

[B4] Bochenek T. Abilova V. Alkan A. Asanin B. de Miguel Beriain I. Besovic Z.. (2017). Systemic measures and legislative and organizational frameworks aimed at preventing or mitigating drug shortages in 28 European and Western Asian countries. Front. Pharmacol. 8 :942. 10.3389/fphar.2017.00942. 29403372 PMC5779072

[B5] Bocquet F. Degrassat-Theas A. Peigne J. Paubel P. (2017). The new regulatory tools of the 2016 health law to fight drug shortages in France. Health Policy 121, 471–476. 10.1016/j.healthpol.2017.03.007 28366432

[B6] Chen S. I. Fox E. R. Hall M. K. Ross J. S. Bucholz E. M. Krumholz H. M.. (2016). Despite federal legislation, shortages of drugs used in acute care settings remain persistent and prolonged. Health Aff. 35, 798–804. 10.1377/hlthaff.2015.1157 27140985 PMC6712565

[B7] De Weerdt E. Simoens S. Casteels M. Huys I. (2015). Toward a European definition for a drug shortage: a qualitative study. Front. Pharmacol. 6:253. 10.3389/fphar.2015.00253 26578963 PMC4626567

[B8] De Weerdt E. Simoens S. Casteels M. Huys I. (2017). Clinical, economic and policy implications of drug shortages in the European Union. Appl. Health Econ. Health Policy 15, 441–445. 10.1007/s40258-016-0264-z 27480539

[B9] Dutch Government, M.o.t.I.a.K.R. (2007). Dutch Medicines Act [Online]. Available: https://wetten.overheid.nl/BWBR0021505 (Accessed July, 2018).

[B10] Dutch Government, Ministry of Health, Welfare Sport. (2017). Report by the Working Party Drug Shortages (in the Netherlands). Available online at: https://www.government.nl/documents/reports/2017/03/30/report-by-the-working-party-drug-shortages-in-the-netherlands (Accessed July, 2018).

[B11] European Directorate for the Quality of Medicines HealthCare (2016). Standard Terms. Available online at: https://standardterms.edqm.eu/ (Accessed September 2016).

[B12] European Medicines Agency (2013). Medicine Shortages. Available online at: http://www.ema.europa.eu/ema/index.jsurl=pages/regulation/general/general_content_000588.jsp&mid=WC0b01ac05807477a5 (Accessed July 2018).

[B13] Fink S. (2016). Drug shortages forcing hard decisions on rationing treatments. The New York Times. January 29, 2016.

[B14] Food and Drug Administration (2015). Permanent discontinuance or interruption in manufacturing of certain drug or biological products. Final rule. Fed Regist 80, 38915–38940. 26155603

[B15] Food Drug Administration (2018). Frequently Asked Questions about Drug Shortages. Available online at: https://www.fda.gov/Drugs/DrugSafety/DrugShortages/ucm050796.htm (Accessed June 1, 2018).

[B16] Fox E. R. Birt A. James K. B. Kokko H. Salverson S. Soflin D. L. (2009). ASHP guidelines on managing drug product shortages in hospitals and health systems. Am. J. Health Syst. Pharm. 66, 1399–1406. 10.2146/ajhp090026. 19635779

[B17] Fox E. R. Sweet B. V. Jensen V. (2014). Drug shortages: a complex health care crisis. Mayo. Clin. Proc. 89, 361–373. 10.1016/j.mayocp.2013.11.014 24582195

[B18] Gray A. Manasse H. R. Jr. (2012). Shortages of medicines: a complex global challenge. Bull. World Health Organ. 90, 158–158a. 10.2471/blt.11.101303 22461706 PMC3314203

[B19] Iacobucci G. (2017). Drug shortages cost NHS pound 38m in November. BMJ 359:j5883 10.1136/bmj.j5883 29263021

[B20] Koba M. (2015). The U.S. has a Drug Shortage and People are Dying. Available online at: http://fortune.com/2015/01/06/the-u-s-has-a-drug-shortage-and-people-are-dying/ (Accessed July, 2018).

[B21] Lynas K. (2013). Patient care and health compromised by drug shortages in Canada: survey of physicians and pharmacists. Can. Pharm. J. 146, 67–68. 10.1177/1715163513482702 23795173 PMC3676197

[B22] Malone C. Acheson J. R. Hinds J. D. McComiskey M. H. (2016). Uterotonics for non-emergent caesarean section: protocol change during UK-licensed drug shortage. Ulster Med. J. 85, 174–177. 27698519 PMC5031104

[B23] McCarthy M. (2013). FDA reveals new initiatives to deal with drug shortages. BMJ 347:f6646. 10.1136/bmj.f6646 24188905

[B24] McCartney M. (2015). Daily drug shortages place avoidable pressure on primary care. BMJ 350:h2514. 10.1136/bmj.h2514 25964678

[B25] Pauwels K. Simoens S. Casteels M. Huys I. (2015). Insights into European drug shortages: a survey of hospital pharmacists. PLoS ONE 10:e0119322. 10.1371/journal.pone.0119322 25775406 PMC4361582

[B26] Quilty S. (2014). Medicines shortages in Australia-the reality. Australas. Med. J. 7, 240–242. 10.4066/AMJ.2014.1933 25031644 PMC4082247

[B27] Rosa M. B. Reis A. M. Perini E. (2016). Drug shortage: a public health problem. Cad Saude Publica 32:e00086916. 10.1590/0102-311x00086916 27783756

[B28] The European Parliament the Council of the European Union (2011). Directive 2001/83/EC of the European Parliament and of the Council of 6 November 2001 on the Community Code Relating to Medicinal Products for Human Use. Available online at: https://eur-lex.europa.eu/eli/dir/2001/83/oj (Accessed July, 2018).

[B29] US Government Accountablility Office (2016). Drug Shortages: Certain Factors Are Strongly Associated with This Persistent Public Health Challenge. GAO (US Government Accountability Office). Available: https://www.gao.gov/products/GAO-16-595 (Accessed GAO-16-595).

[B30] Vail E. Gershengorn H. B. Hua M. Walkey A. J. Rubenfeld G. Wunsch H. (2017). Association Between us norepinephrine shortage and mortality among patients with septic shock. JAMA 317, 1433–1442. 10.1001/jama.2017.2841 28322415

[B31] WHO (2016). Medicines shortages global approaches to addressing shortages of essential medicines in health systems. WHO Drug Inform. 30, 180–185.

[B32] WHO Collaborating Centre for Drug Statistics Methodology ATC - Structure Principles. (2018). Available online at: https://www.whocc.no/atc/structure_and_principles/ (Accessed July 2018).

[B33] Wosinska M. Jensen F. E. V. (2015). Are Shortages Going Down or Not? Interpreting Data From the FDA and the University of Utah Drug Information Service [Online]. Available online at: https://www.healthaffairs.org/do/10.1377/hblog20150408.046227/full/ (Accessed July, 2018).

